# Spatiotemporal Discordance in Five Common Measures of Rurality for US Counties and Applications for Health Disparities Research in Older Adults

**DOI:** 10.3389/fpubh.2015.00267

**Published:** 2015-11-25

**Authors:** Steven A. Cohen, Lauren Kelley, Allison E. Bell

**Affiliations:** ^1^Department of Family Medicine and Population Health, Virginia Commonwealth University, Richmond, VA, USA; ^2^Department of Internal Medicine, Virginia Commonwealth University, Richmond, VA, USA

**Keywords:** rural health, obesity, methods development, elderly population, comparison of methods

## Abstract

**Introduction:**

Rural populations face numerous barriers to health, including poorer health care infrastructure, access to care, and other sociodemographic factors largely associated with rurality. Multiple measures of rurality used in the biomedical and public health literature can help assess rural–urban health disparities and may impact the observed associations between rurality and health. Furthermore, understanding what makes a place truly “rural” versus “urban” may vary from region to region in the US.

**Purpose:**

The objectives of this study are to compare and contrast five common measures of rurality and determine how well-correlated these measures are at the national, regional, and divisional level, as well as to assess patterns in the correlations between the prevalence of obesity in the population aged 60+ and each of the five measures of rurality at the regional and divisional level.

**Methods:**

Five measures of rurality were abstracted from the US Census and US Department of Agriculture (USDA) to characterize US counties. Obesity data in the population aged 60+ were abstracted from the Behavioral Risk Factor Surveillance System (BRFSS). Spearman’s rank correlations were used to quantify the associations among the five rurality measurements at the national, regional, and divisional level, as defined by the US Census Bureau. Geographic information systems were used to visually illustrate temporal, spatial, and regional variability.

**Results:**

Overall, Spearman’s rank correlations among the five measures ranged from 0.521 (percent urban–urban influence code) to 0.917 (rural–urban continuum code–urban influence code). Notable discrepancies existed in these associations by Census region and by division. The associations between measures of rurality and obesity in the 60+ population varied by rurality measure used and by region.

**Conclusion:**

This study is among the first to systematically assess the spatial, temporal, and regional differences and similarities among five commonly used measures of rurality in the US. There are important, quantifiable distinctions in defining what it means to be a rural county depending on both the geographic region and the measurement used. These findings highlight the importance of developing and selecting an appropriate rurality metric in health research.

## Introduction

Rural–urban health disparities in all aspects of health and health care have been realized for several decades, yet the causes, scope, and magnitude of these disparities continue to pose challenges for researchers and policymakers. The environmental, socio-political, cultural, economic, and demographic characteristics of rural America are, in many ways, vastly different from those of urban and suburban areas and present a unique set of circumstances that have implications far beyond research and policy. Understanding the full scope of rural–urban disparities and designing policies, programs, and interventions meant to address them are matters of national priority to ensure health and health care equity for the entire population.

### Rural–Urban Health Disparities

Numerous examples highlight rural–urban health inequalities, including disparities in chronic conditions, health behaviors, and health outcomes. Obesity is a primary contributor to numerous health consequences, but the distribution of obesity is not uniform throughout the US (1). Rural residents are more likely to be obese than their urban counterparts and are also more likely to have chronic diseases related to obesity, such as hypertension and diabetes (2, 3). Compared to their urban counterparts, they are also less likely to engage in protective health behaviors such as increased physical activity and fruit and vegetable consumption (4, 5). This increased prevalence of chronic diseases and decreased likelihood of healthy behaviors are compounded by the fact that rural residents are also less likely to use preventive health services (6).

Rural–urban health disparities are particularly problematic in older adults. Rural older adults, similar to the general rural population, are less likely to visit general practitioners, specialists, and dentists compared to urban residents (6, 7). Furthermore, rural older adults have a higher prevalence of certain chronic disorders when compared to their urban counterparts, even after controlling for other sociodemographic characteristics (8). This, along with a decreased likelihood to visit a physician particularly among older adults, likely leads to the overall poorer health status observed among older rural residents. When compared to their urban counterparts, rural older adults have poorer health status as measured by physical, role, and social functioning, mental health, and health perception (9). Subsequent studies have also found rural–urban differences in the health of cancer survivors (10–12) and overall quality of life in veterans (13, 14) among many others.

Distinct rural–urban patterns were also observed in other aspects of health services utilization in older adults, including use and cost of chiropractic care (15) and medical care for treatment of lower back problems (16). Rural–urban gradients have been observed for preventive health behaviors as well. A recent study showed that as rurality increased, the rate of mammography and colorectal cancer decreased monotonically (17, 18). A related example is the receipt of informal family caregiving to allow older adults to successfully remain in their homes and prevent costly institutionalization that is often harmful to older adults’ well-being and quality of life. Rural informal caregivers to older adults reported worse health and reduced preventive health behaviors than their urban counterparts (19).

### Challenges in Measuring “Rurality”

There is increasing interest among health researchers and policymakers in the community characteristics, such as rurality, that potentially influence health (7, 10–19). Despite the vast use of rurality as an important contextual predictor of differential health outcomes and health services utilization, a common thread in all of these studies is the lack of a universal measure of rurality itself. A wide array of measures exists, each with its own strengths and drawbacks. These include population density, US Census-designated rural and urban status, metropolitan areas, urban influence codes (UICs), rural–urban continuum codes (RUCCs), and Rural-Urban Commuting Area Codes (20). Many of these measures are defined primarily by one or two such community characteristics, like commuting time or influence of nearby urban areas. Some of these measures are continuous measures, while others are dichotomous or ordinal. All have been used in studies of health and medicine to some degree, but there is no consensus on an ideal measure. Furthermore, recent social science research suggests that what defines “rural” or “urban” is context specific (21).

This lack of a universal measure of rurality is manifested in two distinct, but interrelated, ways: finding the optimal geographic unit on which to assess rurality and finding the specific set of characteristics that define rurality. When researchers select an appropriate geographic unit on which to measure rurality, several choices exist, including state, county, zip code, census tract, etc. Each of these, however, has its own benefits and drawbacks (22–24). The central focus of this paper, however, addresses the second challenge: how to measure “rurality” itself and what effect using different measures of rurality will have on assessing health disparities in older adults. While rural–urban gradients in resources and health indicators are well-documented, comparatively little inquiry has been done into how rurality is actually defined and measured, (25, 26) particularly in assessing population characteristics that distinguish rural areas from urban areas (27).

### Objectives

To address this challenge, in this study, we systematically assess the spatial, temporal, and regional differences and similarities among five commonly used measures of rurality in studies of population health in the US. The objectives of this study are: (1) to spatiotemporally describe, compare, and contrast five common measures of rurality among US counties; (2) to assess the internal agreement among the measures for US counties at the regional, divisional, and metropolitan area levels; and (3) to investigate how the prevalence of obesity in the population aged 60+ correlates with rurality for each of the five measures of rurality at the regional and divisional level. We will highlight several key findings of this analysis and its applications for future health research in the development and use of rurality measures.

## Materials and Methods

### Data

To conduct the analysis, data from several sources were first merged to form one large database of county characteristics. Rurality measurements were obtained from the 2010 US Decennial Census and the 1993 and 2003 US Department of Agriculture’s (USDA’s) Economic Research Service. County-level measurements of body mass index (BMI) were abstracted using the 2010 Behavioral Risk Factor Surveillance System (BRFSS) in the population aged 60 years and above. Respondents were classified as “obese” if their BMI were 30 kg/m^2^ or above.

### Measures

Details of the rurality measures are found in Table 1. Four of the most common measurements of rurality were used in this analysis, based on prior literature. Two measures commonly addressed in health research on rurality from the USDA were used: the RUCC (28–31) and the UIC (30–35). For both of these measures, counties are first designated as metropolitan or non-metropolitan, as defined by the federal Office of Management and Budget. “Metropolitan” is often equated with urban areas, while “non-metropolitan” usually refers to more-rural areas. In 2013, there were 1,167 metropolitan and 1,976 non-metropolitan counties in the US. After that designation, the RUCC gives counties a code based on their metropolitan or non-metropolitan status, as well as on population size and adjacency to a metropolitan area, resulting in a nine-part classification (36). Similarly, the UIC forms a classification scheme that also distinguishes metropolitan counties by population size of their metro area, and non-metropolitan counties’ proximity to metro and micropolitan areas and population size of an encompassed city/town, resulting in a 12-part classification (37).According to the USDA, both measures allow researchers to break county data into finer residential groups, beyond metro and non-metro, particularly for the analysis of trends related to population density and metro influence (36, 37). Two of the other most commonly used measures of rurality in the medial literature were abstracted from the 2010 US Census: population density (38) and percent urban population (39). The US Census Bureau identifies and defines two types of urban areas. First, “urbanized areas” are those that contain a population of 50,000 or more. “Urban clusters” have between 2,500 and 50,000 people. In the US, there are 486 urbanized areas and 3,087 urban clusters (27).

**Table 1:** **Five measures of rurality used in the analysis, sources, and number of levels**.

Source	Rurality measure	Type of variable	Distribution	Description
2003 and 2013 USDA	Rural–urban continuum code	Ordinal	12 levels	Based on proximity of metropolitan statistical area and population size, arranged as a continuum
2003 and 2013 USDA	Urban influence code	Ordinal	Nine levels	Based on the estimated economic influence of urban areas on counties and population size
2010 US Census	Population density	Continuous	Right-skewed	County population size divided by county land area
2010 US Census	Percent urban population	Continuous	Right-skewed	US Census definition of percent of county population considered “urban”
2010 US Census	Index of Relative Rurality (28)	Continuous	Approximately symmetric	Composite scale of several component variables. Ranges from 0 to 1

An additional rurality variable was used in this analysis. Unlike the UIC and RUCC, the index of relative rurality (IRR) (40) does not take into account metro boundaries, but instead uses a set of established dimensions of rural–urban characteristics: population, population density, extent of urbanized area, and distance to the nearest metro area. Individually, these measures have been incorporated into many other measures of rurality. The IRR is scaled from 0 to 1, with 0 representing the most urban place and 1 representing the most rural area. However, for the sake of consistency among measures in this analysis, coding was reversed, with 1 indicating the most urban and 0 indicating the most rural.

### Statistical Analysis

The univariate distributions and frequencies were obtained separately for each of the five rurality measures. For the first objective – spatiotemporally describe, compare, and contrast five common measures of rurality among US counties – a bivariate analysis was conducted using Spearman’s rank correlation coefficients between each pair of measurements. Spearman’s rank correlation coefficients were used because all of the five measures incorporated are either continuous or ordinal ranked data with at least nine possible values. This analytical tool can handle assessing potential monotonic associations between pairs of variables that are skewed, ranked, and continuous variables that have a high percentage of the same value (ties) (e.g., percent urban). Descriptive geographic information systems mapping was also used to visually assess the overall spatial and temporal patterns of rurality using each of the five measures.

For the second objective – assess the internal agreement among different rurality measures based on the geographic level of analysis – again, a bivariate analysis was conducted using Spearman’s rank correlation coefficients by Census-designated regions and divisions of the US. Furthermore, an identical correlational analysis was conducted on the Richmond, VA, area to examine the internal consistency among the five measures on a metropolitan area, defined as all counties in which at least 25% of the county land area lies within 40 miles of Richmond city centroid. Surrounding counties were included for analysis if at least 25% of their area were contained within a 50-mile radius of the city.

For the third and final objective – investigate how the prevalence of obesity in the 60+ population correlates with rurality for each of the five measures of rurality at the regional and divisional level – Spearman correlations were examined between percent obese (BMI ≥ 30) in the older population and each of the five rurality measurements. The analysis was conducted nationally and for each Census-designated region and division described in the previous objective. SAS version 9.3 (Cary, NC) was used for all modeling, and ArcMap version 10.1 (Redlands, CA, USA) was used for mapping.

## Results

### Spatiotemporal Consistency of Rurality Measurement

**Figure 1:**
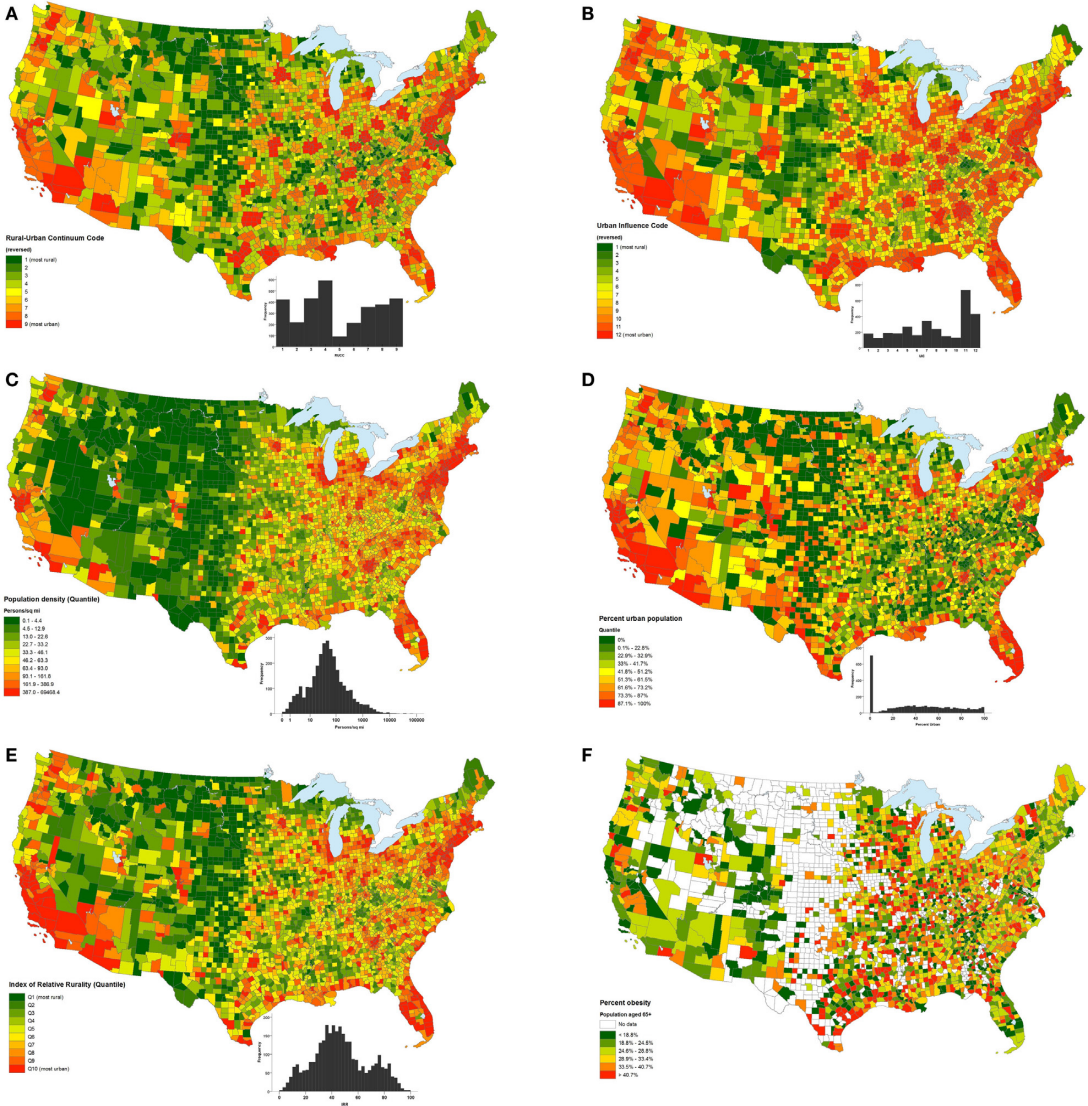
**Geographic distributions by county of rural–urban continuum code (A), urban influence code (B), population density (C), percent urban population (D), Index of Relative Rurality (E), and percent of the 60+ population that is obese (F)**.

The five measures of rurality also varied by statistical and geographic distributions (Figures 1A–E). Population density had an approximately log-normal distribution, and the IRR had a fairly symmetric distribution. The UIC was somewhat uniformly distributed, except at the urban end of the distribution, where higher frequencies were observed. The RUCC also had a nearly uniform distribution. Percent urban had a mixed distribution in which 701 (22.3%) of all US counties had a value of 0 for percent urban, with a generally uniform distribution otherwise. Geographically, the distributions of each of the rurality measures were somewhat similar to each other, with several exceptions. Urban clusters identified by UIC and RUCC tended to be larger than those identified by other measures. Also, urban clusters identified by those two measures appeared as “plateaus” on the maps, indicating that urban areas tended to be broader and more uniformly urban than those urban areas identified by population density, percent urban, and the IRR.

**Figure 2:**
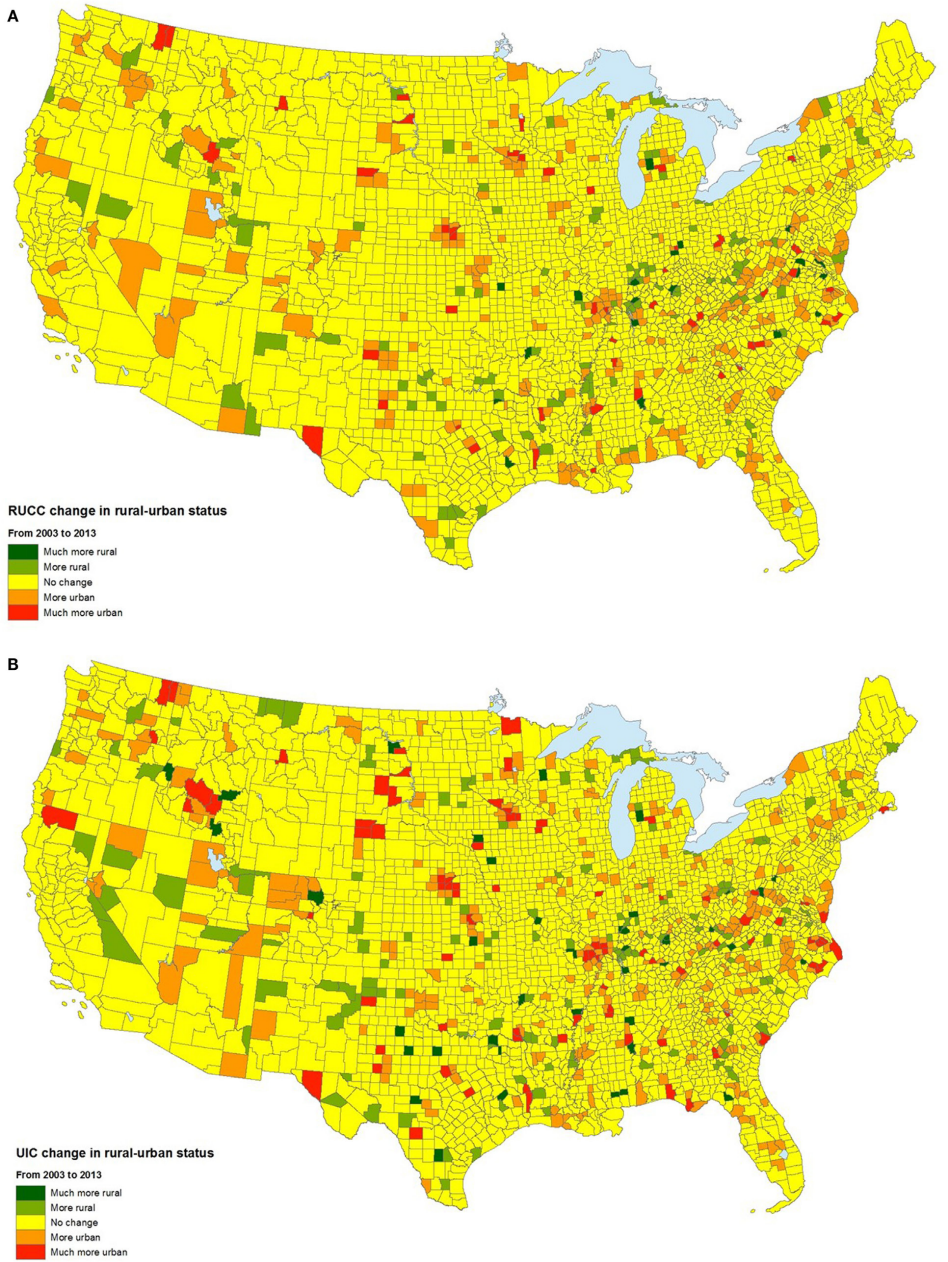
**Temporal changes in the rural–urban continuum code (A) and urban influence code (B), 2003–2013**.

Over time, the spatial distribution of the RUCC remained fairly stable. From 2003 to 2013, 2.653 (85.3%) counties had no change in status based on this variable, 323 (10.4%) became more urban, and 133 counties (4.3%) became more rural, according to the RUCC (Figure 2A). For the UIC, most counties (2,545) did not incur a change in rural–urban designation between 2003 and 2013. Similar to the RUCC, 177 (5.7%) became more rural in that time period, according to the UIC, while 387 (12.4%) became more urban (Figure 2B).

**Figure 3:**
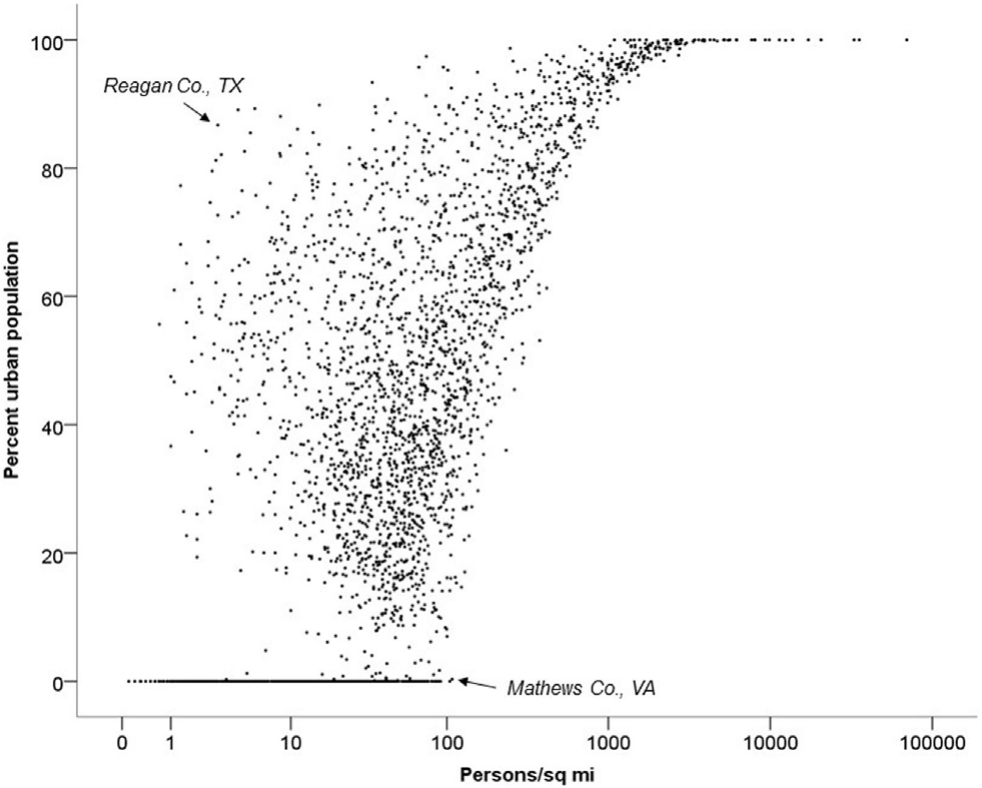
**Association between percent urban population and population density in 2010**.

Percent urban population was least geographically consistent with the other four measures (Figure 3). Generally, counties with higher-percent urban populations were more densely populated, but there were notable exceptions. For instance, in some counties with one or two small cities, percent urban variable was unexpectedly large given a low population density. An example of this includes Reagan County, TX, (noted on figure) with an urban population or 87.1% (90th percentile) and a population density of only 2.9 people per square mile (7th percentile). Other counties have relatively high population densities despite having low urban populations. For example, Mathews County, VA, has an urban population of 0% and a population density of 104.5 people per square mile (73rd percentile).

### Rurality at the Regional, Divisional, and Metropolitan Levels

The correlations among each of the rurality measures differed from the correlations observed at the national level (Table 2) and varied by region (Table 3) and division (Table 4). On the national level, correlations between pairs of rurality measurements were moderate to strong. The correlation between the RUCC and UIC was 0.917, while the correlation between the UIC and percent urban was just 0.521. Similar variability in the level of correlation among the five measures of rurality occurred on the regional level. The correlations between population density and percent urban varied by region. For the entire US, the correlation between these two measures was 0.659, but this correlation for the Northeast, Midwest, South, and West Regions was 0.939, 0.739, 0.658, and 0.788, respectively. At the division level, larger differences in correlation between some measures occurred. For example, the correlation between the UIC and percent urban ranged from only 0.384 in the West South Central Division to 0.802 in the Pacific Division. However, correlations between other measures remained relatively consistent between divisions. For instance, the correlation between IRR and population density remained relatively strong, ranging from 0.811 in the West South Central Division to 0.966 in the Middle Atlantic Division.

**Table 2:** Spearman’s rank correlation coefficients for five measures of rurality*.

	Percent urban	Urban influence code^a^	Rural–urban continuum code^a^	Index of relative rurality
Population density	0.659	0.711	0.746	0.867
Percent urban		0.521	0.659	0.909
Urban influence code^a^			0.917	0.704
Rural–urban continuum code^a^				0.789

*^a^Reverse coding used*.

*All *p*-values were <0.01.

**Table 3:** Spearman’s rank correlation coefficients for five measures of rurality at the Regional level*.

	Percent urban	Urban influence code^b^	Rural–urban continuum code^b^	Index of relative rurality
**Northeast**				
Population density	0.939	0.812	0.834	0.964
Percent urban		0.756	0.782	0.983
Urban influence code^b^			0.968	0.796
Rural–urban continuum code^b^				0.819
**Midwest**				
Population density	0.739	0.751	0.813	0.919
Percent urban		0.538	0.719	0.905
Urban influence code^b^			0.895	0.733
Rural–urban continuum code^b^				0.840
**South**				
Population density	0.658	0.620	0.665	0.829
Percent urban		0.453	0.570	0.941
Urban influence code^b^			0.922	0.598
Rural–urban continuum code^b^				0.691
**West^a^**				
Population density	0.788	0.769	0.825	0.919
Percent urban		0.640	0.792	0.932
Urban influence code^b^			0.896	0.779
Rural–urban continuum code^b^				0.876

*^a^Alaska and Hawaii excluded from analysis*.

*^b^Reverse coding used*.

*All *p*-values were <0.01.

**Table 4:** Spearman’s rank correlation coefficients for five measures of rurality at the Divisional level*.

	Percent urban	Urban influence code^b^	Rural–urban continuum code^b^	Index of relative rurality
**New England**				
Population density	0.935	0.802	0.849	0.954
Percent urban		0.742	0.827	0.980
Urban influence code^b^			0.944	0.792
Rural–urban continuum code^b^				0.857
**Middle Atlantic**				
Population density	0.937	0.803	0.816	0.966
Percent urban		0.737	0.748	0.983
Urban influence code^b^			0.970	0.778
Rural–urban continuum code^b^				0.787
**East north central**				
Population density	0.798	0.687	0.728	0.915
Percent urban		0.535	0.636	0.943
Urban influence code^b^			0.925	0.667
Rural–urban continuum code^b^				0.731
**West north central**				
Population density	0.749	0.715	0.810	0.925
Percent urban		0.500	0.769	0.896
Urban influence code^b^			0.836	0.704
Rural–urban continuum code^b^				0.864
**South Atlantic**				
Population density	0.847	0.612	0.638	0.912
Percent urban		0.514	0.570	0.971
Urban influence code^b^			0.953	0.592
Rural–urban continuum code^b^				0.642
**East south central**				
Population density	0.767	0.514	0.647	0.862
Percent urban		0.404	0.609	0.958
Urban influence code^b^			0.862	0.539
Rural–urban continuum code^b^				0.706
**West south central**				
Population density	0.558	0.691	0.749	0.811
Percent urban		0.384	0.522	0.906
Urban influence code^b^			0.906	0.609
Rural–urban continuum code^b^				0.720
**Mountain**				
Population density	0.728	0.682	0.775	0.883
Percent urban		0.513	0.747	0.917
Urban influence code^b^			0.840	0.692
Rural–urban continuum code^b^				0.846
**Pacific^a^**				
Population density	0.852	0.822	0.860	0.933
Percent urban		0.802	0.865	0.961
Urban influence code^b^			0.945	0.849
Rural–urban continuum code^b^				0.906

*^a^Alaska and Hawaii excluded from analysis*.

*^b^Reverse coding used*.

*All *p*-values were <0.01.

**Figure 4:**
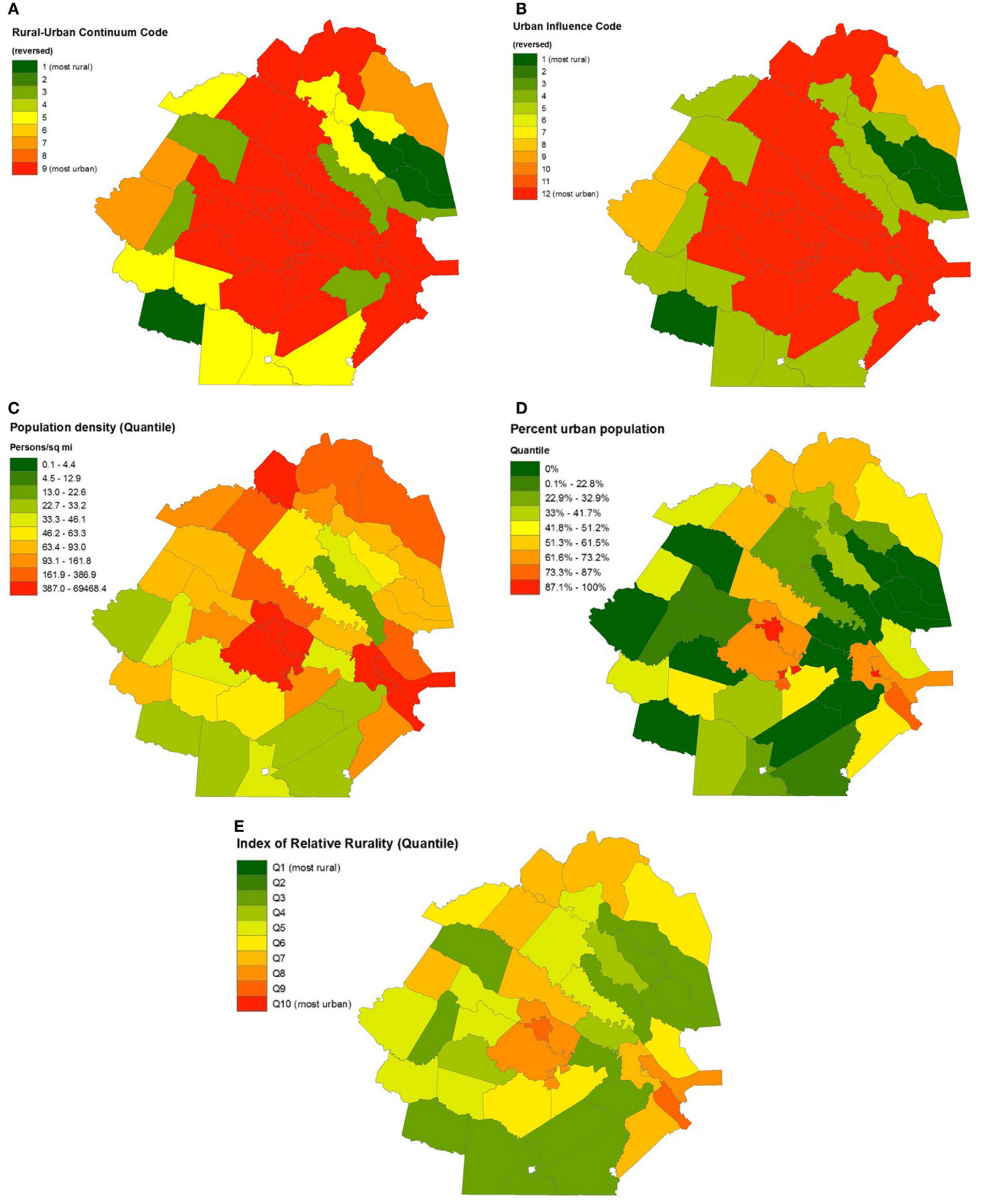
**Geographic distributions of urban influence code (A), rural–urban continuum code (B), percent urban population (C), population density (D), and Index of Relative Rurality (E) in Richmond, VA, USA**.

In the Richmond, VA, USA, metropolitan area, 74% of the 49 counties within 50 miles of Richmond were considered metropolitan (“in metro areas of 1 million or more residents”) in both the UIC and the RUCC (Figure 4). Within this region, there were notable discrepancies among the five measures of rurality. For instance, 4 of the 13 Richmond-area counties with a 0% urban population were considered the most urban, according to the UIC. Nearly half (15) of the 31 counties classified as “most rural,” according to the UIC, had urban populations of at least 60%. Spearman correlations between pairs of measures were similar to those of the Census divisions. The highest correlation occurred between the RUCC and UIC (0.989), while the weakest correlation was observed between percent urban and UIC (0.544).

### Application: Five Rurality Measures and Obesity

The spatial distribution of obesity in the population aged 60 and above by county is found in Figure 1F. Spearman correlations between percent obese and rurality (Table 5) are generally weak, but vary by geographic level and individual measure of rurality. For the entire US, percent obesity prevalence was associated with percent urban population (rho = −0.044, *p* = 0.034), but was not significantly associated with any of the other four rurality measures. On the regional level, obesity was negatively and significantly associated with four of the five measures (all but UIC) of rurality in the Northeast, while obesity was positively and significantly associated with four of the five rurality measures (all but population density) in the West. Notable variability in the obesity–rurality association occurred among divisions in the same region. For instance, obesity was significantly and negatively associated with all five measures of rurality in the Middle Atlantic Division states, but not significantly associated with any rurality measure in the New England Division. In the South Region, none of the rurality measures were significantly associated with obesity prevalence if you look at the region as a whole. However, in the South Atlantic Division within the South Region, obesity prevalence was significantly and positively associated with population density (rho = −0.122, *p* < 0.01), but not any of the other four rurality measures. In the West South Central Division, only IRR was associated with obesity prevalence, and the association was positive (rho = 0.160, *p* < 0.001).

**Table 5:** **Spearman’s rank correlation coefficients for obesity five measures of rurality for the entire US, and by Census Region and Division**.

	RUCC	UIC	Population density	Percent urban	IRR
US	−0.008	−0.003	−0.005	−0.044*	0.016
Northeast Region	−0.148*	−0.121	−0.216**	−0.219**	−0.151*
New England Division	−0.042	−0.015	−0.178	−0.157	−0.107
Middle Atlantic Division	−0.280**	−0.255**	−0.301**	−0.315**	−0.248**
South Region	−0.018	−0.026	−0.035	0.008	0.024
South Atlantic Division	−0.048	−0.055	−0.122**	−0.061	−0.063
East South Central Division	−0.049	−0.067	0.008	0.052	0.021
West South Central Division	0.055	0.058	0.085	0.092	0.160**
Midwest Region	0.007	0.015	−0.012	−0.037	0.005
East North Central Division	0.016	0.034	−0.019	0.015	0.024
East South Central Division	−0.011	−0.017	−0.030	−0.110	−0.048
West Region	0.175**	0.156*	0.077	0.134*	0.145*
Mountain Division	0.160	0.137	0.051	0.197*	0.173*
Pacific Division	0.112	0.085	−0.032	0.009	0.038

*p < 0.05.

**p < 0.01.

## Discussion

The internal agreement between rurality measures varied widely based on geographic location. For instance, population density and percent urban were strongly correlated in the Northeast Region (*r* = 0.939) and less correlated in the South Region (*r* = 0.658). Likewise, at the divisional level, the East South Central and South Atlantic Divisions consistently showed some of the lowest internal agreements among all measures, while the New England and Middle Atlantic regions consistently showed some of the highest internal agreements among measures.

The picture of varying strength of internal agreement among measures based on geographic location becomes clearer as a “snapshot” is taken at the city level. For instance, when comparing the counties surrounding the city of Richmond (South Region, South Atlantic Division) with those surrounding Providence (Northeast Region, New England Division) in terms of the UIC and RUCC, it would appear that these cities are similar. In both cities, more than 89% of counties have both a UIC value and a RUCC value, indicating that they are metropolitan in nature. However, upon comparing percentage urban and population density, it becomes apparent that these two cities are quite different. The counties surrounding Providence consistently tended to display characteristics indicative of an urban setting in addition to the UIC and RUCC, with median values for population density, percent urban, and IRR all above the 90th percentile for the entire US. For example, the median population density of the Providence area was 985 people per square mile (95th percentile), and the median percent urban was 90.0% (92nd percentile). However, in Richmond, the additional measures tell a different story from that of the UIC and RUCC, one with a greater degree of rurality. The median population density and percent urban was only 58.2 people per square mile (57th percentile) and 17% (27th percentile), respectively, showing that the surrounding counties are likely more rural than the UIC and RUCC codes would indicate.

Of all the measures, the one that consistently appeared to demonstrate a truer picture of rurality across cities was the IRR. Richmond had IRR values close to the median value for the entire country. These results are more consistent with what one would anticipate when treating rurality as multi-dimensional, as does the IRR. These varying degrees of consistency across measures dependent on geographic location highlight the importance of not only considering which measure to use based on the specific research question, but also on the geographic location in which the analysis is taking place. While all five measures seem to have relatively strong and consistent internal agreement in New England and the Northeast, the comparatively weak internal agreement seen particularly in the Southern portion of the US highlights the need to think more closely about which measure to use in the event analysis is concentrated in one region of the US. These observations are illustrative of non-stationarity across space, which is tied to “local” spatial analysis in other studies of rural–urban health inequities.

The above-mentioned pitfalls are important to consider and are further exemplified by the observed agreement between each of the measures and a health outcome that has been shown to have a well-established link to an individual’s rural–urban living status. It has been well-documented that rural residence is associated with an increased prevalence of obesity, yet there has been a lack of consistency between studies when measuring rurality. Our findings, in particular, exhibit how the associations between rurality and health outcomes in older adults with an established rural–urban disparity and can vary based on the measure used to assess rurality and the geographic location and level at which analysis is performed.

As observed with the agreement between rurality measures, the observed relationship between obesity and rurality varies depending not only on the rurality measure used, but also on the geographic location. Furthermore, as observed, the decision to use one rurality measure over another could have a substantial impact on observed relationships. The fact that the obesity–rurality relationship varied by both the rurality measurement used and the geographic location suggests that the concepts of “rural” and “urban” are both multi-dimensional, and their impact on health varies by location. These concepts are described further in the sociological literature.

Several important limitations of this analysis need to be considered. First, the analysis was conducted on the county level, largely due to the availability of data at that geographic level. Therefore, it was not possible to compare the five measures within counties, despite the potential for counties, especially ones encompassing a large geographic area, to be heterogeneous with respect to rurality. In addition, counties are not consistent in terms of both size and function from state to state. Enormous heterogeneity exists in the size of counties across the US, with west of the Mississippi on average far larger, and the range of size variation can be as high as 10,000-fold. Consider the example used in this study of the Richmond, VA, metropolitan area. In Virginia, major cities of varying population size are themselves considered equivalent to counties and are independent of nearby counties. Richmond, for example, is an independent city and is not incorporated into any other county in the state. The same holds true for even smaller cities in Virginia. However, in many other states, such as the northeastern ones, cities are located within counties that often contain other cities and towns. Therefore, in a state such as Virginia, small independent cities that contain relatively small populations are considered statistically “equivalent” to comparatively much more heavily populated and geographically larger counties in other areas of the country that include many cities and towns of varying population sizes.

The analysis is subject to two statistical limitations as well. First, no geospatial analysis was performed; each county was treated as an independent unit of observation in this analysis. Counties in closer proximity are more likely to share sociodemographic and cultural characteristics than counties that are further apart. Spatial dependence may account for some of the observed associations (41) and can be addressed in future studies. Compounding this issue is that many of the rurality measures themselves are dependent upon nearby characteristics, such as proximity to nearest metropolitan or urbanized area (e.g., RUCC, UIC, and IRR). The impact of rurality on health may, therefore, be much greater in highly rural areas near an urbanized area, compared to similar rural areas that are hundreds of miles from the nearest urbanized area. This disparity in distance from urbanized areas may explain some of the regional and divisional inconsistencies among the five measures. Second, only monotonic associations could be observed using the Spearman correlations in this study. In other words, non-monotonic associations, such as a J-shaped or a U-shaped association between rurality and obesity, might result in a weak or null association when simply examining the rank correlation as was done in this study. Lastly, another important limitation to consider is that only one health outcome – obesity – was assessed. Future research could examine how each of these measurements distinctly and perhaps uniquely influences different aspects of population health metrics and could examine potential non-linear associations between health and rurality.

Despite these limitations, this study is among the first such analysis to systematically assess the spatial, temporal, and regional differences and similarities among five commonly used measures of rurality in studies of population health in the US. There are important, quantifiable distinctions in defining what it means to be a rural county depending on both the geographic region and the measurement used. The findings of this analysis underscore the importance of developing and selecting an appropriate rurality metric in health research and represent an important first step in understanding the similarities and differences among rurality measurements available to health researchers.

## Author Contributions

SC conceived the project, wrote the initial draft of the manuscript, and conducted part of the data analysis. LK created maps, provided editorial comments, and assisted with data visualization. AB wrote subsequent versions of this manuscript and assisted with the regional analysis.

## Conflict of Interest Statement

The authors declare that the research was conducted in the absence of any commercial or financial relationships that could be construed as a potential conflict of interest.
